# Seeing a Work of Art Indirectly: When a Reproduction Is Better Than an Indirect View, and a Mirror Better Than a Live Monitor

**DOI:** 10.3389/fpsyg.2019.02033

**Published:** 2019-09-10

**Authors:** Marco Bertamini, Colin Blakemore

**Affiliations:** ^1^Visual Perception Laboratory, Department of Psychological Science, University of Liverpool, Liverpool, United Kingdom; ^2^School of Advanced Study, Centre for the Study of the Senses, University of London, London, United Kingdom

**Keywords:** art, aesthetics, aesthetic experience, perception, museum

## Abstract

Visiting a museum and seeing an original artwork can be a special experience. We use a survey and a set of hypothetical questions to explore how such experience would be affected by changes in how the artwork is seen. In a first study, participants imagined that they had traveled to see a painting that they particularly like. They discover that it is impossible to directly see the original painting. Three alternatives are offered: seeing an optical reflection (using a mirror), seeing a video screening (a closed-circuit camera), or seeing a reproduction. In all cases, it is made clear that the size, brightness, and resolution will match that of the original. In addition, these options could be within the same room as the original, in the room next door, or in a different building. Results show that physical distance did not affect significantly the responses. However, there was an overall preference for seeing a reproduction as opposed to an optical or digital image. Contrary to the idea that the original is always superior to a copy, many people felt that a direct view of a copy is a preferable experience than an indirect view. The second study was focused directly on the comparison between a mirror and a monitor. Here we highlighted the fact that for the mirror light coming from the mirror originated from the painting. Data were collected in Britain and in China. In both cases, there was a clear preference for the mirror over the monitor.

## Introduction

Art plays an important role in society. We can see this by the production of artworks early in human history and by the large number of people that every day go to museums and exhibitions. Some even regard art as a pinnacle of human culture (Zaidel, 2010; Pelowski et al., 2017a), and Pelowski et al. (2017b) found a correlation between what was classified as a work of art and liking. The study of the aesthetics experience has also remained central to the interest of scholars in psychology and neuroscience over many decades (Arnheim, 1974; Kubovy, 1986; Ramachandran and Hirstein, 1999; Chatterjee, 2004; Leder et al., 2004). In a famous lecture in 1934 (“Art as Experience”), the philosopher John Dewey argued that what is important is not the material aspects of the work of art, but the process in its entirety, and in particular the experience of art (Leddy, 2016).

In this study, we asked participants to evaluate the impact of not been able to see a work of art (a painting) directly. It is accepted that the experience of seeing an original artwork depends on context. Some locations provide the expected home for art, and confer value to the experience, as in the case of theaters, cinemas, and museums. For paintings, a museum may create a quiet and thoughtful environment, sometimes characterized using the metaphor of a white cube (O’Doherty, 1986; Gartus and Leder, 2014). The popularity of art exhibitions and museums is strong in many countries. For example, in 2016, in the USA, museums were attended more than major league sporting events and amusement parks put together (as cited in Fingerhut and Prinz, 2018).

Another factor in the experience of art is the link with the artist through the material nature of the artwork. When people visit the Louvre and see Leonardo’s *Mona Lisa*, they are present in front of the very canvas on which Leonardo worked for many years of his life (Lorusso and Natali, 2015). Similarly, when entering Leonardo’s house in Amboise, France, visitors are aware that they are walking within the corridors and rooms in which the great artist lived during the latter part of his life. Newman and Bloom (2012) studied the special value of original artworks. They concluded that people assess art objects on the basis of the unique creative act (performance) and also in relation to the physical contact with the original artist (contagion).

To reflect on the role of knowledge about whether a work of art is the original, we consider the case of the Lascaux Cave. This cave was discovered in September 1940 by a teenager (named Ravidat) while looking for his dog (named Robot). The cave walls are covered with depictions of animals, and the complex was opened to the public after the war, in 1948. It was closed in 1963 when it became clear that the carbon dioxide, heat, and humidity were harmful to the images.

In 1983, Lascaux II was opened. This is a copy of part of the cave complex (the Great Hall of the Bulls and the Painted Gallery) a few hundred meters away from the cave location. Despite the fact that this is a copy, Lascaux II is the most visited Paleolithic site in the world. It is in itself a work of art, which took almost a decade to complete. The painter Monique Peytral used the same methods and materials as the original artists. She copied the original design by projecting photos of the drawings onto the walls and painting over them.

Lascaux III is an 800 m^2^ exhibition and it has been traveling the world since 2012. More recently, in 2016, President Hollande inaugurated Lascaux IV. This is a replica designed by Norwegian architectural firm Snøhetta and located at the foot of the hill. To optimize the experience, this site includes a soundtrack of Ravidat whistling for Robot, and an environment in which temperature, air pressure, and damp smell are similar to that of the cave at the time of discovery.

Lascaux therefore provides a range of examples of how to experience art. The original (which we cannot see) is on rocks and these objects and images are shared with artists from the Magdalenian period (17,000–12,000 years ago). Lascaux II was created by an artist to be as faithful as possible and in proximity of the original. Lascaux III attempts to bring the cave around the world. Finally, Lascaux IV is a technologically state-of-the-art twenty-first century replica, possibly enhanced with respect to the original. A single work of art has now been multiplied into different experiences, which may be difficult to compare.

When discussing Lascaux with friends, we observed that some were enthusiastic about the experience of a visit to Lascaux II, while others felt that there was no reason to travel and see it as it was only a copy. This range of views (from a wonderful experience, to something worthless) is remarkable and was part of the motivation for our study.

## Identity and Authenticity

Modern technology offers multiple ways of gathering sensory information about people and objects. For example, to what extent is having a conversation with a person over a videolink any different from a conversation in person? In the case of art, this opens up questions about how artworks are experienced and the importance of the physical presence of the object.

In philosophy, issues of authenticity overlap with issues of identity and ontology. In some cases, philosophers have resorted to thought experiments. The most famous such experiment dates back to ancient Greece, and is about Theseus ship (the best known version is in Plutarch, but the idea was debated by Heraclitus and Plato, and later by Thomas Hobbes and John Locke). Imagine that the ship is kept in a temple. Over time some parts need repair, so they are replaced. When all parts of the ship are removed and replaced by new parts, is the new object still the ship of Theseus? (Nozick, 1981; Pickup, 2016).

The philosopher Currie (1985) has suggested that there are conditions under which a copy is as aesthetically valuable as the original. This is called the transferability thesis. Writing in particular about paintings, the argument is clearly stated: “there is an aesthetically relevant difference between the two if and only if there actually is a perceptible difference between them” (p. 155). Currie also explicitly says he is not considering the complex issue of forgery and deception, and likewise we will avoid this additional aspect.

There are practical challenges in running a study that compares actual works of art with reproductions, but there have been some in the literature. For example, Locher et al. (1999) asked museum goers at The Metropolitan Museum of Art (New York City) to evaluate nine original paintings. Some participants rated instead slides of the artworks, and another group saw images on a computer screen. Some ratings were higher for the originals, but responses were largely similar for the three formats. Locher et al. (1999) concluded that this evidence lends support to the idea that art experience may be transferable, and that observers may make allowances for the limitations of a medium. In a subsequent study, this result was confirmed with untrained participants (Locher et al., 2001).

Several studies in the literature have demonstrated the importance of context in aesthetic appreciation, interest, and liking. The museum setting positively contributes to these aspects of art experience (e.g., Specker et al., 2017). Brieber et al. (2015) and Grüner et al. (2019) confirmed the museum effect (compared to a lab setting). In addition, they tested the effect of genuineness by comparing real artworks (paintings) hanging on walls with reproductions of these artworks (PowerPoint presentation). They also found that the responses were similar.

In psychology, the term essentialism has been used to refer to the tendency to believe that categories (e.g., women, chairs, Picasso paintings) have an underlying true nature. Essentialism provides a reasoning heuristic in children and adults (Gelman, 2003). For example, children make different inferences about kids described as “kids who eat carrots” and those described as “carrot eaters” (Gelman and Heyman, 1999) and they are unwilling to accept an identical replacement for an attachment object (Hood and Bloom, 2008). Essentialism suggests that people may perceive a famous painting as having a true nature, and that this nature is lost in a copy. In 2017, the philosopher Jesse Prinz suggested that if the *Mona Lisa* burned in a fire, people may prefer to see the ashes than a copy (Prinz, 2017). This may be a case of essentialism as the essence of an object may remain within a physical object even when the qualities of the object change.

## Survey About a Hypothetical Museum Experience

**Figure 1:**
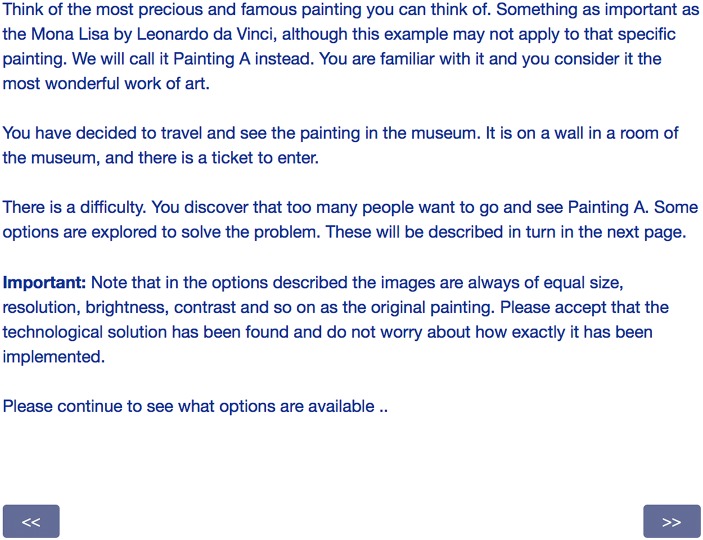
The scenario presented to all participants in the first study.

We studied self-reported preferences for different ways to see a painting. Using an online questionnaire, we presented participants with a scenario (Figure 1) and asked them to evaluate the impact of not been able to see a work of art directly. The methodology is similar to that of a philosophical thought experiment (as in Theseus ship) but with the difference that it allows the gathering of responses from a large sample. The survey was advertised on the university website and on social media and through personal contacts. Therefore, although in theory anybody could access the survey, a large proportion of participants are likely to have been psychology students and their friends and family. There was never any payment or reimbursement as part of the survey.

The study was approved by the Health and Life Sciences Committee on Research Ethics (Psychology, Health and Society) of the University of Liverpool (reference 0734).

Think of the most precious and famous painting you can think of. Something as important as the Mona Lisa by Leonardo da Vinci, although not that specific painting. We will call it Painting A instead. You are familiar with it and you consider it the most wonderful work of art.

You have decided to travel and see the painting in the museum. It is on a wall in a room of the museum, and there is a ticket to enter.

There is a difficulty. You discover that too many people want to go and see Painting A. Some options are explored to solve the problem. These will be described in turn in the next page.

**Important***: Note that in the options described the images are always of equal size, resolution, brightness, contrast, and so on as the original painting. Please accept that the technological solution has been found and do not worry about how exactly it has been implemented.*

**Figure 2:**
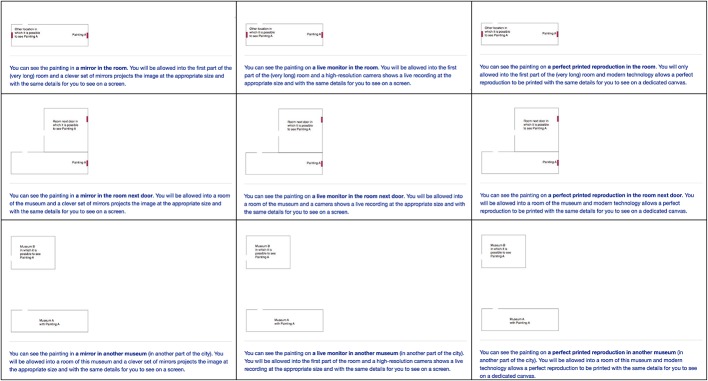
The three columns show the three questions presented to an individual (in random order). The three rows show the three locations that were presented to different groups of individuals. Small maps were included in the survey to illustrate the locations. These were the same room as the original, the room next door, or a room in another building.

Participants were offered the option to see Painting A in three ways: in a mirror, on a live monitor, or on a reproduction (three columns of Figure 2). These three possibilities were presented in random order.

You can see the painting in a mirror in the room. You will be allowed into the first part of the (very long) room and a clever set of mirrors projects the image at the appropriate size and with the same details for you to see on a screen.

You can see the painting on a live monitor in the room. You will be allowed into the first part of the (very long) room and a high-resolution camera shows a live recording at the appropriate size and with the same details for you to see on a screen.

You can see the painting on a perfect printed reproduction in the room. You will be allowed into the first part of the (very long) room and modern technology allows a perfect reproduction to be printed with the same details for you to see on a dedicated canvas.

The three rows of Figure 2 show conditions shown to different groups. The difference was the location, the painting (in a mirror, a monitor, or as a reproduction) was to be seen in the same room, in a room next to the room with the original, or in another building.

For each scenario and situation, participants were asked the following question:

The museum is considering what ticket price to charge in this case.

In your personal case would you be happy to:

- pay the same as the ticket for seeing the painting in the traditional way (no discount)
- would not consider this alternative at all
- pay a discounted ticket which is ___ % of the full ticket (for instance if you say 80% and the full ticket was 100 pound then the discounted ticket would cost 80 pound).

In addition to what they would prefer, they were then asked the following similar question (with the same options):

In your opinion, most people would be happy to:

- pay the same as the ticket for seeing the painting in the traditional way (no discount)
- would not consider this alternative at all
- pay a discounted ticket which is ___ % of the full ticket (for instance if you say 80% and the full ticket was 100 pound then the discounted ticket would cost 80 pound).

It is already been found in the literature that people are more critical when evaluating according to their own standards (Leder et al., 2016).

Next, they were asked a question about the importance of knowing that the painting was not the original. The wording was as follows:

Despite the fact that the painting is not seen directly as an original object, imagine a slight variation to the situation described above. Suppose that the way the painting is shown (with a mirror) is such that it is impossible to tell the difference compared to the original.

In your personal case if you believed that you are seeing the original, do you think that your experience would be:

- the same as seeing the original
- somewhat diminished because the original is not actually in front of me
- completely worthless because the original is not actually in front of me.

The words in brackets in this example (“with a mirror”) matched the scenario, and therefore could refer to a monitor or to a reproduction.

Finally, this question was asked with respect to other people:

In the case of most people, if they believed that they are seeing the original, do you think that their experience would be:

- the same as seeing the original
- somewhat diminished because the original is not actually in front of me
- completely worthless because the original is not actually in front of me.

Two-hundred and forty-six participants completed the survey (169 females). Average age was 30.7 years (SD = 13.87). We also asked about their experience with art. Few were professional artists (*N* = 6) and a minority said that they were artists although not at a professional level (*N* = 51). Other items at the beginning of the survey collected information about age, education level (Vocational, GCSE, High school or A-Level, University degree, Master’s degree, Doctorate, None of the above), and when was the last time that they had visited a museum. Because the study was advertised online, the participants were not exclusively students. Indeed, approximately half (128) had an undergraduate degree (or above). We recoded this variable as dichotomous (University-educated vs. non-University educated).

## Results (Main Study)

Participants were assigned randomly to one of three scenarios (different locations). Therefore, the size of the subgroups was similar (same room = 81, next door = 89, other building = 76). The main question was about what people would do when offered the alternative options.

**Figure 3:**
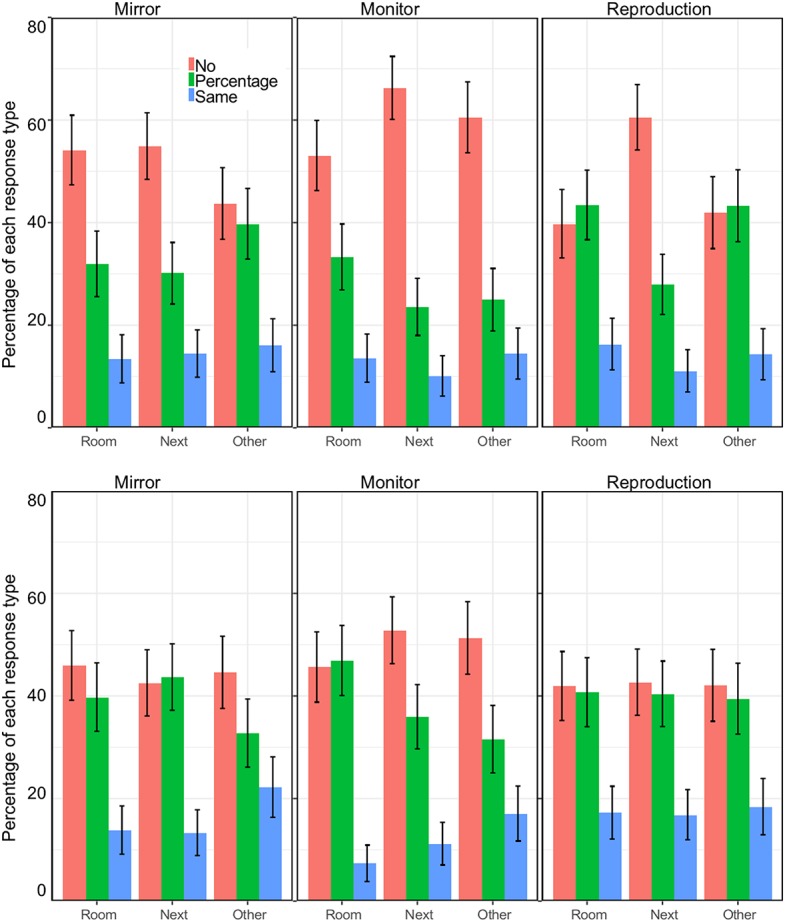
Percentages for the three types of responses (no discount because it would be worthless, a discount, and the same as the original) as a function of location. The columns show the three media (mirror, monitor, and reproduction) and the two rows show the response when the question is about the person, and when the question is about what most people would do. Error bars are standard errors for the mean.

Overall, about half of the respondents said they would not consider the option described: 51.6% for the mirror, 60.2% for the monitor, and 49.2% for the reproduction. A minority said they were happy and did not need any discount: 14.6% for the mirror, 12.6% for the monitor, and 14.2% for the reproduction. The presence of these two large groups is interesting as it suggests a range of views, including both extremes (Figure 3). When the question was about what most people would say, responses were similar. In about half of the cases, participants predicted that other people would not consider the option as acceptable: 44.7% for the mirror, 50.0% for the monitor, and 42.7% for the reproduction. A minority predicted that most people would be happy and would not request any discount: 16.3% for the mirror, 11.8% for the monitor, and 17.9% for the reproduction.

Note a shift in the values in the case of a question about most people, as if the respondents were using a stricter stance for themselves and expected other people to be more willing to accept the option offered. The frequencies of the two responses (would not consider it at all, would pay the same amount) were different [mirror: 𝝌^2^(1) = 49.7, *p* < 0.001; monitor: 𝝌^2^(1) = 75.1, *p* < 0.001; reproduction: 𝝌^2^(1) = 46.3, *p* < 0.001]. However, the association between type of response and whether the question was about the self or about most people was not confirmed [mirror: 𝝌^2^(1) = 0.66, *p* = 0.42; monitor: 𝝌^2^(1) = 0.07, *p* = 0.79; reproduction: 𝝌^2^(1) = 1.65, *p* = 0.19].

To fully analyze the data, including the percentage of the price of the ticket, we created a new variable. We coded the choice as 0 if they would not consider at all the option (not willing to see the painting under those conditions). We coded it as 100 if they were happy to pay the original price, and we used the percentage to express their willingness to get a ticket. Therefore, we have a number between 0 and 100% that is our proxy for how much they valued that particular option. We call this Ticket value.

Overall, the mean values were 32.9% (SD = 38.9) for the mirror, 26.0% (SD = 37.3) for the monitor, and 34.1% (SD = 38.8) for the reproduction. For the question about most people, the values were similar: 36.6% (SD = 38.4) for the mirror, 30.3% (SD = 36.3) for the monitor, and 38.6% (SD = 39.4) for the reproduction.

**Figure 4:**
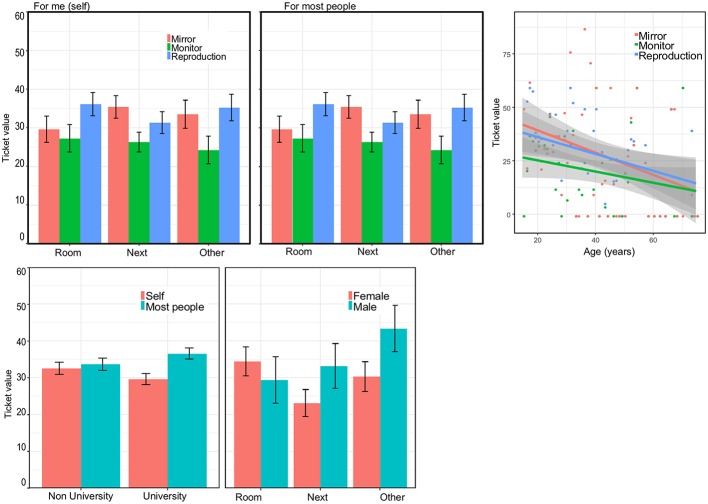
Average Ticket value. This score combines the three possible responses (no, discount, full price) into a single percentage between 0 and 100%. Top left: value as a function of location and medium. Top right: value as a function of age (log scale) and medium. Bottom left: Interaction between level of education and whether the question was about that individual or about most people. Bottom right: interaction between location and sex. Error bars are standard errors for the mean.

We used a mixed ANOVA with the following within-subjects factors: Medium (mirror, monitor, reproduction) and Person (self, most people), and the following between-subjects factors: Location (same room, next door, other building), Sex (male, female), Art experience (not an artist, an artist), and Education (University educated or not). As there were only few professional artists we included professional and non-professional artists in a single group. We also included age as a continuous covariate. Average values are shown in Figure 4. It is evident that how much people were happy to pay increased with age; however, this was not the focus of our study and entering age as a covariate allows us to test for other factors controlling for age.

There was a significant main effect of Medium [*F*(2,221) = 3.75, *p* = 0.024, $\eta_{p}^{2}$ = 0.017] and of Person [*F*(1,221) = 4.32, *p* = 0.039, $\eta_{p}^{2}$ = 0.019]. The polynomial contrast for Medium confirmed that the value increased linearly from Monitor, to Mirror, to Reproduction [*F*(1,221) = 7.80, *p* = 0.006, $\eta_{p}^{2}$ = 0.034]. For Person, values were higher when the question was about the self. The continuous variable Age was also significant [*F*(1,221) = 6.24, *p* = 0.013, $\eta_{p}^{2}$ = 0.027].

There was an interaction between Person and Education [*F*(1,221) = 5.37, *p* = 0.021, $\eta_{p}^{2}$ = 0.024]. For non-university educated participants, responses were similar when the question was about the self and when it was about others. By contrast, university-educated participants had lower value for what they were willing to pay themselves and higher value for what most people would pay.

No other effects or interactions were significant. Of the non-significant results, there was a trend worth mentioning for the interaction between Sex and Location [*F*(2,224) = 2.88, *p* = 0.058, $\eta_{p}^{2}$ = 0.025]. For males, it seems that scores increase for locations farther away from where the original painting is located (same room, next doors, other museum). For females, the highest score is in the same room as the original (Figure 4, right panel). We must be careful not to over interpret this; however, one possibility is that males feel more strongly about being in the same room as people who can see the original. This would create a form of public discrimination between groups: those who can and those who cannot see the original, something perceived as a source of humiliation.

**Figure 5:**
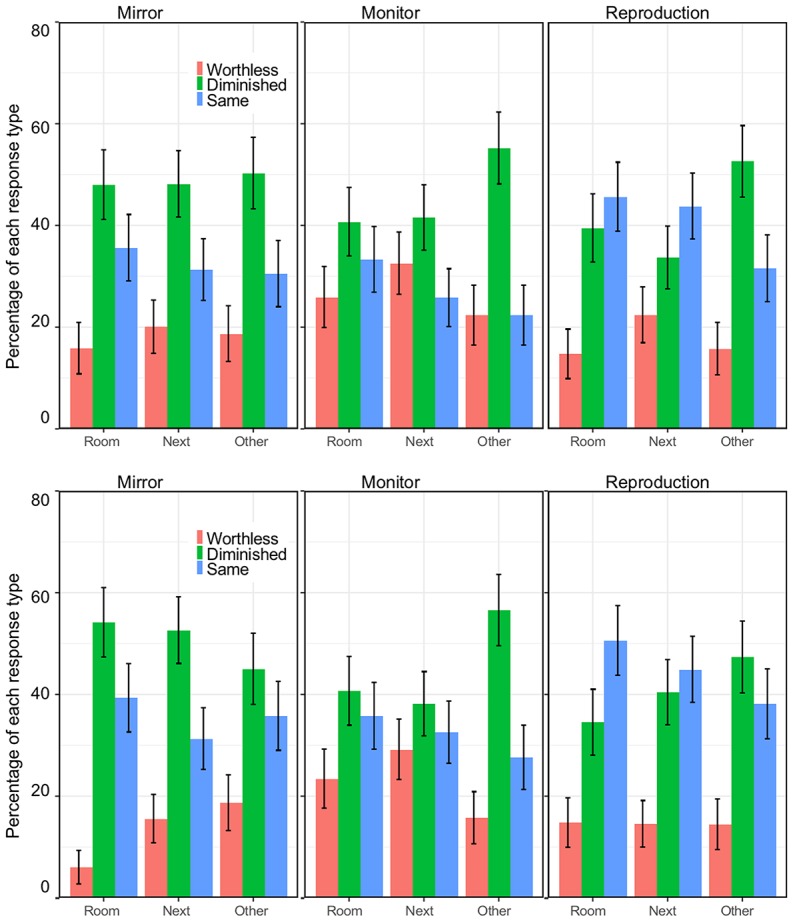
Percentages for the three types of responses (it would be worthless, the experience would be diminished, and the same as the original) as a function of location. These data are for the question about what one would expect if they do not know that the painting is not the original. The columns show the three media (mirror, monitor, and reproduction) and the two rows show the response when the question is about the self, and when the question is about what most people would do. Error bars are standard errors for the mean.

We turn to the question of how people responded when asked to imagine that they believed that the object was the original. The most striking result is the range of views, many people thought that the experience would be worthless (21.1%), but a third thought that it would be the same (33.4%) (see Figure 5). The pattern was similar when the question was about how most people would respond (second row). In this case the belief that the experience would be worthless was expressed by fewer people (17.1%), and more people thought that it would be the same (37.4%). This trend is consistent with what was observed in the previous analysis: participants expected other people to be more willing than themselves to consider the experience as acceptable or equivalent to seeing the original. The frequencies of the two extreme responses (worthless, the same) were different for mirror: [𝝌^2^(1) = 9.24, *p* < 0.002] and for reproduction [𝝌^2^(1) = 21.0, *p* < 0.001] and not for monitor [𝝌^2^(1) = 0]. However, the association between type of response and whether the question was about the self (the respondent) or about most people was not confirmed [mirror: 𝝌^2^(1) = 2.04, *p* = 0.15; monitor: 𝝌^2^(1) = 1.78, *p* = 0.18; reproduction: 𝝌^2^(1) = 1.26, *p* = 0.26].

## Second Study

Some results from the first study were clear. The least valued way to see a painting was by a digital device (video camera and monitor). We were surprised by the fact that mirrors were not chosen as a good way to see the image by more people. We reasoned that a mirror should provide a potential link with the actual painting as the light bouncing from the painting itself eventually reaches the eye of the person looking at the mirror. We worried that the wording of the scenario may have not conveyed the special process of how light if reflected and travels from painting to eye. Indeed, the wording made the mirror appear similar to the monitor. Therefore, we conducted a second survey focused on the comparison between mirror and a video camera combined with a monitor. Since location had no major effect in main study we only described the scenario in the same room.

This second study had also an additional motivation. Although the first study collected data online and we know that there was a range of people taking part from many countries, and a range of ages, the majority were students and academics in Britain as this is the target group to whom the study was advertised. Moreover, the language of the survey was English. In the second survey, we had an English version, targeted to undergraduate students in England, and a version in Mandarin, targeted to undergraduate students in China. We are interested in the generality of our findings across languages and cultures.

**Figure 6:**
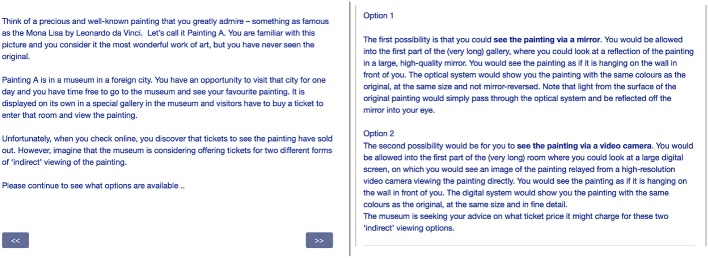
The scenario described in the second study, and the two options presented in the next page. The order of the two options was randomly chosen per participant.

Using an online questionnaire, we asked participants to consider the impact of not been able to see a painting directly. They were presented with two options (see Figure 6), and told that the museum is seeking their advice on what ticket price it might charge for these two “indirect” viewing options.

Think of a precious and well-known painting that you greatly admire – something as famous as the Mona Lisa by Leonardo da Vinci. Let’s call it Painting A. You are familiar with this picture and you consider it the most wonderful work of art, but you have never seen the original.

Painting A is in a museum in a foreign city. You have an opportunity to visit that city for one day and you have time free to go to the museum and see your favorite painting. It is displayed on its own in a special gallery in the museum and visitors have to buy a ticket to enter that room and view the painting.

Unfortunately, when you check online, you discover that tickets to see the painting have sold out. However, imagine that the museum is considering offering tickets for two different forms of ‘indirect’ viewing of the painting.

Option 1

The first possibility is that you could see the painting via a mirror. You would be allowed into the first part of the (very long) gallery, where you could look at a reflection of the painting in a large, high-quality mirror. You would see the painting as if it is hanging on the wall in front of you. The optical system would show you the painting with the same colors as the original, at the same size and not mirror-reversed. Note that light from the surface of the original painting would simply pass through the optical system and be reflected off the mirror into your eye.

Option 2

The second possibility would be for you to see the painting via a video camera. You would be allowed into the first part of the (very long) room where you could look at a large digital screen, on which you would see an image of the painting relayed from a high-resolution video camera viewing the painting directly. You would see the painting as if it is hanging on the wall in front of you. The digital system would show you the painting with the same colors as the original, at the same size and in fine detail.

The two options were presented in different order for different respondents (randomly). The participant was asked to answer a question identical to that used in the first study about what they would consider a reasonable ticket to pay.

## Results (Second Study)

A total of 360 people completed the Mandarin version of the survey, and 200 people completed the English version. Participants were assigned randomly to one order (mirror first = 280, monitor first = 280). The sample was larger than that of the first study, which is useful to compare the two languages (English and Mandarin). However, participants were mainly undergraduate students, with a mean age of 21.2 (SD = 7.5) and 23.2 (SD = 4.08) for English and Mandarin versions respectively. Thus, we do not have the opportunity of testing the role of education.

**Figure 7:**
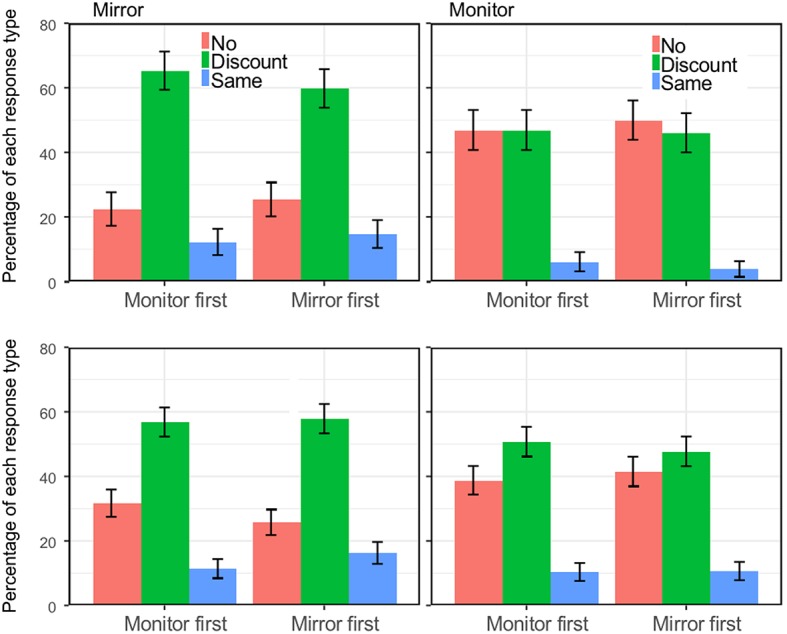
Percentages for the three types of responses (no discount because it would be worthless, a discount, and the same as the original) as a function of Order of question (Monitor first, Mirror first) and separately for Medium (Mirror and Monitor) and for Country (Britain, China). Error bars are standard errors for the mean.

**Figure 8:**
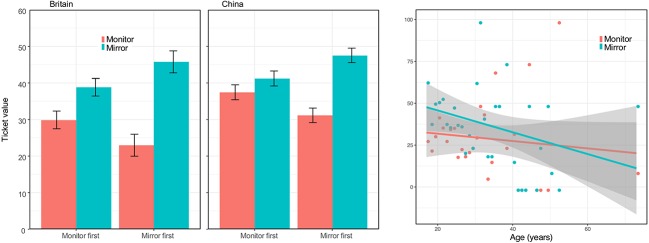
Percentages for the three types of responses (no discount because it would be worthless, a discount, and the same as the original) as a function of Order of question (Monitor first, Mirror first) and separately for Medium (Mirror and Monitor) and for Country (Britain, China). (top right) Value as a function of age (log scale) and medium. Error bars are standard errors for the mean.

For the mirror, more than a third of the respondents said they would not consider the option described: 35.4%. A minority said they were happy and did not need any discount: 10.6%. For the monitor, 22.19% would not consider this option and only 3.82% would pay the same (Figure 7). Overall, the pattern is not completely different from the first study except for a much larger proportion of people who opted for the discount. To fully analyze the responses, we computed the Ticket value in which all three responses are combined. These values are shown in Figure 8.

We used Ticket value as dependent variable in a mixed ANOVA. The within-subjects factor was the Medium (Mirror or Monitor). The between-subjects factors were the Order of the questions, sex, and language (English or Mandarin). To keep things as similar as possible to the analysis of the first study, we also included Age as a covariate.

There was an effect of Medium [*F*(1,551) = 8.72, *p* = 0.003, $\eta_{p}^{2}$ = 0.016]: the Ticket value was higher for the Mirror than for the Monitor. There was also an interaction between Medium and Order [*F*(1,551) = 6.71, *p* = 0.010, $\eta_{p}^{2}$ = 0.012]. As one can see in Figure 8, the interaction is due to higher values for whichever option is presented first. There was also an effect of Age [*F*(1,551) = 6.16, *p* = 0.013, $\eta_{p}^{2}$ = 0.011]. Ticket value decreases with age, as it was observed in the first study. We followed up the interaction effect with two *post hoc* tests to test if the Ticket value was higher for the Mirror condition in each of the two Order conditions. This was confirmed [*t*(279) = 2.50, *p* = 0.013 and *t*(279) = 7.92, *p* < 0.001, for Monitor first and Mirror first, respectively].

The results of the second study are consistent with those of the first study. Seeing the painting in the mirror was judged as a preferred option compared to a digital image of the painting. This was a within rather than a between design and presentation order also affected preference, with a primacy effect. In both studies what people were willing to pay decreased with age. One clear novelty of the second study was the comparison of results from a study in English, taken mainly by British undergraduates, and a version in Mandarin, taken mainly by Chinese undergraduates.

## Discussion

Using a survey, we collected views about how the experience of seeing a painting is affected by the way in which the image is made visible. In particular, we compared an indirect view by means of a mirror, and indirect view by means of a video camera and screen, and a direct view of a reproduction. In all cases, we made it very clear that the image had the same size, color, brightness and resolution. We asked to consider a hypothetical scenario where it is not possible to see the original work of art, and as a way to rate the alternative options, we asked to say how much cheaper the price of the visit should be.

Within these hypothetical scenarios, people expressed a range of opinions. It is interesting that there were large numbers of responses at both extremes. For many, it was not worth seeing the painting in any way other than seeing the original, and they did not consider any discount as adequate. However, for others, it was acceptable to pay the same ticket as the people who could see the original painting directly even if they could only see it indirectly or could only see a reproduction. Very different views therefore coexist in the population. Anecdotally, this is also true for the Lascaux Cave discussed in the introduction. Although large numbers of visitors enjoy Lascaux II and IV, some people would not consider traveling to the location of the cave to then only see a reproduction.

Our participants were stricter in the evaluation of what they themselves would find acceptable, compared to what they expected instead for “most people” (see also Leder et al., 2016). The answers to this second question were more tolerant of the options offered. The percentage of the ticket price that they would pay, overall, was only 30.6%, while what they expected for most people was 35.0%. Note these average values are low because they include the 0% from the cases in which they would not consider the option.

Next, we consider the issue of the medium. We compared a mirror reflection, a closed-circuit video camera (monitor), and a reproduction. Here, there was a pattern across the population with preference for the reproduction as compared to the digital (video) medium. The response to the optical (mirror) option was intermediate. We expected a superiority of the mirror compared to a monitor, but we did not expect a preference for the reproduction, which is a different object altogether with respect to the original. Indeed, based on an essentialist heuristic, we expected the copy to be liked the least.

The mirror in particular is an experience similar to a direct view, given the compelling visual experience that people have when seeing mirror reflections, including the image of their own body (Maravita et al., 2002; Bertamini et al., 2011; O’Sullivan et al., 2017).

In study one, we were surprised that the mirror condition was not considered the most valuable. One possibility is that being so close to the actual artwork and yet only being able to see its reflection made people aware of and unhappy with the constraint. This aspect (seeing the original but only indirectly) may not have played so much of a role in the case of a reproduction. We still have to keep in mind that responses varied considerably between individuals and also with sex. When opinions vary so much, the wording of the question is also critical, we can see that in the comparison between study one and study two. In the second study, we highlighted the fact that the light reflected comes from the artwork and that may have given the mirror an advantage over the monitor.

The fact that the preferred option was the reproduction may reflect the value that people assign to the presence of the material canvas, even though in this case it is a copy, as opposed to an indirect view. It is possible that an indirect view, through a mirror or a digital system, may feel less similar to a visit to see the painting. If the view is indirect, perhaps it is not different enough from seeing the painting on television or in a photo, which people can do without traveling to the location where the painting is kept.

In a second study, we compared the mirror and the monitor, and compared an English version with mainly participants from Britain, and a Mandarin version with participants from China. Results confirmed that the mirror was preferred to the monitor, but there was no difference between English and Mandarin groups.

Our methodology focused on choices and behavior, that is, what participants would do about the options. Only the additional question about the importance of knowing that the painting was not the original asked to evaluate the experience. Despite its limitations, hypothetical scenarios can be useful also to study metacognition about aesthetic value and aesthetic emotions. These terms are, however, still debated in the literature (Leder et al., 2004; Marković, 2012; Menninghaus et al., 2019).

In summary, questions about how the experience of seeing a painting is affected by seeing the original, or a reflection, a video, or a copy are not answered unanimously. For some people, not seeing the original is worthless, for others it is perfectly acceptable. Surprisingly, a copy is not always worse than an indirect view, on the contrary it may be the best option (first study) and a mirror reflection is better than an image shown using a video camera and a monitor (second study). Despite the large individual differences, the type of responses were similar in two different cultures (Western sample and Chinese sample).

## Data Availability

The datasets generated for this study are available on request to the corresponding author.

## Ethics Statement

The studies involving human participants were reviewed and approved by Health and Life Sciences Committee on Research Ethics (Psychology, Health and Society). The patients/participants provided their written informed consent to participate in this study.

## Author Contributions

MB collected the data and wrote the report. MB and CB conceived the original idea and completed the manuscript.

### Conflict of Interest Statement

The authors declare that the research was conducted in the absence of any commercial or financial relationships that could be construed as a potential conflict of interest.
